# Prevalence of Hepatitis C Virus Infection in General Population of Mashhad, Northeastern Iran

**Published:** 2017-03

**Authors:** Sanaz AHMADI GHEZELDASHT, Mohammad Reza HEDAYATI-MOGHADDAM, Khosro SHAMSIAN, Farhad FATHIMOGHADAM, Hamid Reza BIDKHORI, Seyed Abdolrahim REZAEE

**Affiliations:** 1. Research Center for HIV/AIDS, HTLV and Viral Hepatitis, Academic Center for Education, Culture and Research (ACECR), Razavi Khorasan Branch, Mashhad, Iran; 2. Immunology Research Center, Inflammation and inflammatory Diseases Division, School of Medicine, Mashhad University of Medical Sciences, Mashhad, Iran

**Keywords:** HCV infection, Prevalence, General population, Iran

## Abstract

**Background:**

Hepatitis C Virus (HCV) infection is one of the major blood-borne infections worldwide. HCV carriers may develop chronic hepatitis leading to liver cirrhosis and hepatocellular carcinoma (HCC). There is no overall estimate of the infection prevalence in the northeast of Iran. We have performed this research in order to determine accurately the prevalence and risk factors of HCV infection among general population in Mashhad.

**Methods:**

During 2009, 1678 people between 1 to 90 yr old with the mean age of 29.1±18.5 yr were selected randomly by multistage sampling from different geographical regions of the city proportionate to sex and age distribution of population in 2006 census. ELISA was used to screen for antibodies and RT-PCR tested the positive samples.

**Results:**

HCV infection was detected in 7/1654 cases; overall prevalence of the infection was 0.42% (95%CI: 0.17–0.87%), 0.80% and 0.11% among males and females, respectively (*P*= 0.051). One HCV-infected subject was also positive for hepatitis B surface antigen (HBsAg), however, no cases showed HIV or HTLV seropositivity.

**Conclusion:**

In comparison with similar studies, the prevalence of HCV infection in Mashhad is low.

## Introduction

HCV infection is a serious health problem in both developed and developing countries (1). Globally, 150–200 million people, ∼3% of the world’s population, are living with chronic hepatitis C (2, 3) and more than 350000 people die every year from HCV-related liver diseases (4).

The reported prevalence of HCV infection varies greatly among the countries. The infection rates were estimated high (>3.5%) in Central and East Asia, North Africa and the Middle East; are intermediate (1.5%–3.5%) in South and Southeast Asia, sub-Saharan Africa, Central and Southern Latin America, Caribbean, Oceania, Australasia and Central, Eastern and Western Europe; and are low (<1.5%) in Asia-Pacific, Tropical Latin America and North America (3). In our region, the reported prevalence varied greatly; 0.8% in Kuwait, 2.4% in Turkey, 3% in Pakistan and 8.7% in Azerbaijan (5).

The first report on the prevalence of HCV infection in Iran was presented, as 0.3% among blood donors (6). The prevalence of HCV infection among general population in different provinces of the country is from 0.3% to 1.6% (5, 7, 8). On the other hand, the prevalence of HCV infection among individuals at risk was much higher than normal population. HCV infection prevalence was reported among hemodialysis and thalassemic patients as high as 25% (9).

HCV strains are classified into six distinct virus genotypes (1 to 6) and more than 70 subtypes (e.g., subtype 1a, 1b) (10). The distribution of HCV genotypes varies from country to country; genotypes 1, 2 and 3 are distributed widely around the world (11, 12). In Iranian HCV patients, the most common subtypes of HCV were found to be 1a (47%) and 3a (36%) (13).

Mashhad with population of 2.3 million people (census 2006), the second main city in Iran, located near the geographical border of Afghanistan and Turkmenistan. Mashhad as a holly city for Muslims attracts more than 20 million tourists and pilgrims every year. A recent study on 382 HCV-infected patients demonstrated that genotypes 3a (40.0%) and 1a (39.2%) are the predominant genotypes in Mashhad (14).

We have previously demonstrated that Mashhad remains an endemic area for HTLV-I infection (15) and prevalence of HBV infection in this city is slightly lower than that of the nation (16). This community-based study was aimed to determine the prevalence of HCV infection in general population of Mashhad, Northeastern Iran.

## Methods

In this cross-sectional study, 1678 subjects were selected randomly by multistage cluster sampling methods from all of 12 municipality areas and 40 districts of Mashhad, Northeastern Iran during May and Sep 2009. We tried to include equal ratios of the both sexes as well as 10 percentiles for the age according to the 2006 census. The sampling method was described in details elsewhere (16).

A questionnaire about demographic data as well as possible risk factors was completed. Blood samples were taken after obtaining an informed consent and the sera were stored at −20 °C. The samples were assessed initially for anti-HCV by enzyme-linked immunosorbent assay (ELISA) using the EIAgen HCV Ab (v.4) kit (Adaltis Italia S.p.A., Italy) according to the manufacturer’s instruction. Reactive Samples further analyzed using in-house qualitative nested reverse transcriptase-polymerase chain reaction (RT-PCR) for HCV. Extracted RNA with commercial kits (viral RNA mini kit, Qiagen, Germany) was reverse-transcribed using a Revert Aid TM H minus First Strand cDNA Synthesis kit (Fermentas, Germany) according to the manufacturer’s instruction. Table 1 shows sequences of primers for PCR and its protocol. There was no possibility to assay HCV genotyping in the study period.

**Table 1:** The nucleotide sequences of primers and PCR protocols

**Nucleotide position**	**Sequence (5′–3′)**	**PCR protocol**
HCV f1	CATAGATCACTCCCCTGTGAGG	94 °C for 3 min and 20 cycles, 94 °C for 40 sec 58 °C for 40 sec, 72 °C for 40 sec final extension at 72 °C for 5 min
HCV r1	GGCGGTTGGTGTTACGTTTGGT	
HCV f2	CCCTGTGAGGAACTACTGTCTTC	94 °C for 3 min, 30 cycles, 94 °C for 40 sec 58 °C for 40 sec, 72 °C for 40 sec final extension at 72 °C for 5 min
HCV r2	GGTGCACGGTTACGAGACCTC	

f: forward, r; reverse

ELISA techniques for detection of anti-HTLV (Dia.pro, Italy) and anti-HIV (GB, Taiwan) antibodies as well as the surface antigen of hepatitis B virus (HBsAg) (Radim, Italy), according to manufacturers’ instructions, also tested positive HCV samples.

Data were described as mean ± SD or frequencies and 95% confidence interval (95% CI) was given for the infection frequency.

## Results

Overall, 1678 people aged 1–90 yr with the mean age of 29.1±18.5 yr were included. Demographic data of the participants were shown in Table 2.

**Table 2:** Socio-demographic data of study population from Mashhad, Iran

**Variable**	**Number**	**%**
Sex		
Male	763	45.5
Female	915	54.5
Age (yr)		
<=5	134	8.0
6–11	156	9.3
12–16	159	9.5
17–20	184	11.0
21–24	181	10.8
25–29	182	10.8
30–34	136	8.1
35–44	193	11.5
45–54	164	9.8
>=55	189	11.3
Marital status (people older than 14)		
Single	789	48.3
Married	787	48.1
Divorced/ Widowed	59	3.6
Literacy (people older than 5)		
Illiterate	114	7.5
Primary school	347	22.8
Secondary	289	19.0
High School/ Diploma	498	32.8
Academic education	271	17.9
Employment (people older than 14)		
Employed	497	35.9
Unemployed	888	64.1
Family income (million Rials per month)		
< 3	814	51.1
3–5	553	34.7
>5	226	14.2
Ethnic background		
Fars	1444	86.8
Turk	101	6.1
Afghani	62	3.7
Others	56	3.4
Place of birth		
Razavi Khorasan Province	1402	84.9
Other provinces in Iran	222	13.4
Other countries (Afghanistan, Iraq)	27	1.6

### Prevalence of HCV infection

Twenty-four persons refused to have blood taken and were excluded from subsequent analyses. Among 1654 subjects, anti-HCV seropositivity was detected in 11 cases (0.67%, 95% CI: 0.33–1.19%), of whom seven samples were further confirmed by RT-PCR technique. Overall, prevalence of HCV infection was 0.42% (95% CI: 0.17–0.87%), 0.80% and 0.11% among males and females, respectively (*P*=0.051). Demographic and risk factor data for HCV-seropositive subjects were shown in Table 3. One HCV-infected subject (no. 5) was also positive for HBsAg, however, no cases showed HIV or HTLV seropositivity.

**Table 3:** Demographic and risk factor data for HCV-seropositive patients from Mashhad, Iran

***No.***	***Sex***	***Age (yr)***	***Marital status***	***Education (yr)***	***Occupational status***	***Race***	***Risk factor***	***HCV antibodies***	***HCV RNA***
1	Male	3	−	−	−	Arab	None	+	+
2	Male	10	Single	< 6	−	Fars	None	+	+
3	Male	24	Single	6–8	Self -employed	Fars	Dentistry procedure, Hospitalization, Tattooing	+	+
4	Male	36	Single	< 6	Unemployed	Fars	Hospitalization, Drug abuse ^*^	+	+
5	Male	36	Single	12	No reported	Fars	Dentistry procedure, Tattooing	+	+
6	Male	44	Married	6–8	Self -employed	Fars	Dentistry procedure, Hospitalization	+	+
7	Female	20	Single	14	No reported	Fars	None	+	+
8	Male	9	−	< 6	−	Fars	Dentistry	+	−
9	Male	21	Single	14	Academic student	Fars	None	+	−
10	Female	14	Single	10	School student	Fars	Dentistry	+	−
11	Female	2	−	−	−	Fars	None	+	−

* Based on methadone therapy,

′+′; positive,′−′; negative

## Discussion

As the first population-based study on the HCV prevalence in Northeastern Iran, we found that the overall prevalence of the infection in Mashhad was 0.42%. The overall prevalence of HCV infection in the general population of Mashhad was 0.13% with RT-PCR method in 2010–11 (17). This difference in the prevalence might be due to the different demographic features of the studied population. Merat et al. reported a significantly higher prevalence of HCV infection in males compared to females especially in young and middle-aged groups (7). In the current study, nearly equal proportion of the both genders was included and ten percentiles by age were established according to reported population of the city in 2006 census. However, the participants in Shakeri et al. study were much older than our population (39 vs. 29 yr) and male to female ratio was 0.49 (17).

The low rate of HCV infection in our population is compatible with reports from other studies in the country. A systematic review included eight reports published from six provinces during 1998–2006, calculated that the HCV infection prevalence rate in Iran is 0.16% (95% 95% CI:0%–0.59%), the prevalence rate varied from zero among pregnant women in Khuzestan to 1.3% among residents of nursing home in Guilan (5). Moreover, in a study performed on 5684 subjects from three provinces of Iran during 2006, the overall weighted prevalence of anti-HCV was 0.5%; the lowest and highest rates were observed among samples from Tehran (0.3%) and Hormozgan, Southern Iran, (1.6%), respectively (7). On the other hand, a survey of 5235 people older than 15 yr who live during 2014 in Birjand City, South Khorasan, reported a 0.2% and 0.14% prevalence rate for HCV infection by ELISA and PCR tests, respectively (18). Of 12 (0.2%) of 6145 participants from general population of urban and rural areas of Amol, Mazandaran showed a seroactivity for HCV and according to PCR analysis, the prevalence of true HCV infection was 0.05% (19).

The prevalence of HCV infection in the general population varies considerably in different regions of Iran. The reason for the dissimilarity in HCV infection rates is not clear but could be due to the differences in the lifestyles, habits, and rates of high-risk behaviors as well as quality of public health services in different geographic regions (20). Moreover, different confirmatory methods used for ELISA-positive cases (recombinant immunoblot assay, HCV-RNA PCR or both tests) could be considered as a probable reason for this variation.

In the current population study, HCV infection rate was lower than those reported from neighboring countries of Iran were. In comparison with similar studies, the prevalence of HCV infection in Iran is low (5). In Azerbaijan, northern neighbor to Iran, a high rate of infection with hepatitis C (8.7%) was revealed (21). Similarly, in Pakistan a high prevalence of hepatitis C; 4.95%, 3.64% and 1.72% among adult, young and pediatric populations respectively was reported (22). Furthermore, the frequency of anti-HCV positivity among 1157 randomly selected patients attending an outpatient clinic in Istanbul, Turkey, was reported as high as 2.4% (23).

Donor selection procedures, as well as improvement in blood transfusion services, have been emphasized to minimize the adverse results of blood transfusion (24, 25). According to national prevention strategies, screening and treating high-risk groups such as intravenous drug abusers and multi-transfused patients should be strongly accomplished (24, 26).

## Conclusion

The HCV prevalence in our population is lower than many reports from other countries in the region. The low rate of HCV infection among our population could be partly to some national programs such as blood safety measures or those targeted high-risk population. In Iran, screening of blood donations for HBsAg, HIV and HCV started from 1974, 1989 and 1996, respectively. HCV rate among Iranian blood donors has declined from 0.3% in 2001 to 0.12% in 2007.

## Ethical considerations

Ethical issues (Including plagiarism, Informed Consent, misconduct, data fabrication and/or falsification, double publication and/or submission, redundancy, etc) have been completely observed by the authors.
